# 上皮间充质转化：肺癌侵袭转移及耐药性的生物学基础与临床展望

**DOI:** 10.3779/j.issn.1009-3419.2025.102.07

**Published:** 2025-03-21

**Authors:** Hengxing SUN, Mengting XIONG, Shuanshuan XIE, Jing WEN

**Affiliations:** ^1^200072 上海，同济大学附属第十人民医院呼吸科; ^1^Department of Respiratory Medicine, Shanghai Tenth People’s Hospital, Tongji University School of Medicine, Shanghai 200072, China; ^2^200331 上海，同济大学医学院; ^2^Tongji University School of Medicine, Shanghai 200331, China; ^3^200433 上海，同济大学附属上海市肺科医院结核科; ^3^Department of Tuberculosis, Shanghai Pulmonary Hospital, Tongji University School of Medicine, Shanghai 200433, China; ^4^200072 上海，同济大学附属第十人民医院党委办公室; ^4^Office of the CPC Committee, Shanghai Tenth People's Hospital, Tongji University School of Medicine, Shanghai 200072, China

**Keywords:** 肺肿瘤, 上皮间充质转化, 侵袭, 转移, 耐药性, Lung neoplasms, Epithelial-mesenchymal transition, Invasion, Metastasis, Drug resistance

## Abstract

肺癌是全球癌症死亡的首要原因，具有高发病率和死亡率的特征。肿瘤的侵袭与耐药是肺癌患者治疗失败的首要原因，尤其是化疗药物耐药及表皮生长因子受体（epidermal growth factor receptor, EGFR）突变靶向治疗耐药，严重影响了晚期肺癌患者的治疗效果。上皮间充质转化（epithelial-mesenchymal transition, EMT）作为一种重要的生物学过程，与组织胚胎发育、器官生成、损伤修复及肿瘤侵袭等生理或病理过程密切相关。众多研究表明EMT通过多种信号通路的介导，在肺癌的发生、发展和转移中起重要作用，同时与肺癌细胞的耐药性密切相关。因此，针对EMT相关的分子机制及病理生理的研究有助于逆转肺癌药物治疗的耐药性，改善患者的预后。本文结合国内外相关文献，综述了EMT在肺癌侵袭转移与耐药性中的研究进展。

## Abstract (en)

Lung cancer is the leading cause of cancer-related deaths worldwide, characterized by high incidence and mortality rates. The primary reasons for treatment failure in lung cancer patients are tumor invasion and drug resistance, particularly resistance to chemotherapeutic agents and epidermal growth factor receptor (EGFR) mutant targeted therapy, which considerably undermine the therapeutic outcomes for those with advanced lung cancer. Epithelial-mesenchymal transition (EMT) serves as a crucial biological process closely associated with physiological or pathological processes such as tissue embryogenesis, organogenesis, wound repair, and tumor invasion. Numerous studies have indicated that EMT, mediated through various signaling pathways, plays a pivotal role in the initiation, progression, and metastasis of lung cancer, while it is also closely associated with drug resistance in lung cancer cells. Therefore, research focusing on the molecular mechanisms and pathophysiology related to EMT can contribute to reversing drug resistance in drug treatment for lung cancer, thereby improving prognosis. This article reviews the progress in research on EMT in the invasion, metastasis, and drug resistance of lung cancer based on relevant domestic and international literature.

肺癌作为全球范围内癌症相关性死亡的首要原因，其高发病率和死亡率是亟待解决的重大挑战。尽管近年来在肺癌的早期诊断、手术治疗、放疗、化疗以及分子靶向治疗等方面取得了显著进展，但肺癌患者的总体预后情况仍不容乐观，尤其是晚期肺癌患者表现出高复发率与低生存率，大大加重了家庭与社会的疾病负担^[1]^。其中，肺癌的侵袭转移能力和对治疗的耐药性是导致患者预后不良的关键因素。

上皮间充质转化（epithelial-mesenchymal transition, EMT）作为一种重要的生物学过程，在胚胎发育、组织修复以及肿瘤进展等多个方面发挥着至关重要的作用^[2,3]^。近年来，越来越多的研究开始关注EMT在肺癌侵袭转移和耐药性中的作用。研究^[4,5]^发现，EMT不仅参与了肺癌的发生和发展过程，还与肺癌细胞对化疗药物和靶向治疗药物的敏感性降低密切相关。因此，深入探究EMT与肺癌侵袭转移及耐药性的关系，对揭示肺癌的发病机制、开发新的治疗策略以及改善患者的预后具有重要的意义。

本文旨在对EMT调控肺癌侵袭转移及耐药性的研究进展作一综述，探讨当前研究中存在的问题和未来可能的研究方向，为肺癌的临床治疗提供新的思路和策略。

## 1 EMT

### 1.1 EMT的基本概念与生物学特征

EMT是指上皮细胞经过一系列的生物化学改变，失去自身特性而转化为间充质细胞的形态学过程^[6]^。该转化过程对原肠胚形成、神经嵴形成及器官生成等组织胚胎发育事件具有重要意义^[7]^。越来越多的研究^[6,8]^指出，上皮细胞经EMT转化为间充质细胞，参与组织修复、器官纤维化与肿瘤侵袭转移等病理过程。与EMT相反的过程即间充质上皮转化（mesenchymal-epithelial transition, MET），与EMT类似地发生在组织胚胎发育、组织创伤修复及癌症发生、发展过程中^[2,3]^。

EMT的过程伴随着原始上皮细胞的形态学、分子学以及生物学功能的多重改变（图1）。上皮细胞通过其基底面与基底膜相互作用，在形态上由鹅卵石样的多边形变成较为细长的纺锤形或梭形的间充质细胞。EMT的激活伴随着细胞极性丧失、细胞连接破坏、上皮基底膜结构改变以及细胞外基质重组，转化后的间充质细胞失去极性，细胞骨架发生改变^[9]^。在此过程中，与上皮细胞极性及其细胞连接相关的分子，如E-钙黏蛋白（E-cadherin）、上皮细胞黏附分子（epithelial cell adhesion molecule, EpCAM）、紧密连接蛋白ZO-1（zona occludens-1, ZO-1）、闭合蛋白（Occludin）、紧密连接蛋白（Claudin）、细胞角蛋白19（cytokeratins 19, CK19）等表达水平减少，而与间充质细胞相关的表达标志物被激活，如N-钙黏蛋白（N-cadherin）、波形蛋白（Vimentin）、纤维连接蛋白及β1/β3整合素等^[9,10]^。随着细胞极性消失与黏附分子的改变，细胞间的黏附度降低，其迁移与运动特性增强，细胞获得侵袭能力^[11]^。

**图1:**
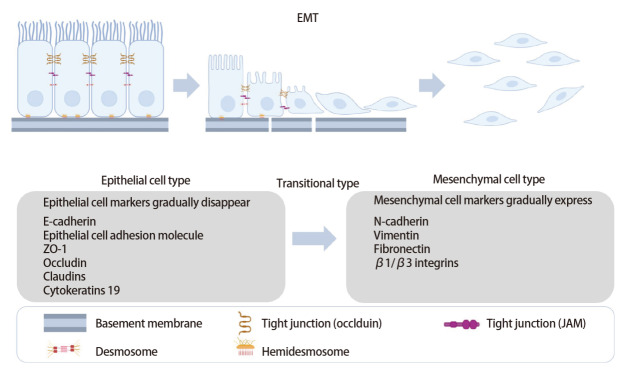
上皮间充质转化的过程。呈多边形的上皮细胞逐渐转化成纺锤形或梭形的间充质细胞，细胞极性丧失，细胞连接破坏，细胞骨架改变，上皮-基底膜结构改变。在分子水平，上皮细胞的标志物（E-钙黏蛋白、上皮细胞黏附分子等）逐渐消失，间充质细胞的标志物（N-钙黏蛋白、波形蛋白等）表达逐渐增加。

### 1.2 EMT的分类

自1982年Greenburg和Hay提出EMT后，其已被证实在胚胎发育、组织再生、肿瘤侵袭等病理过程中发挥作用^[12]^。目前，依据其发生的生物学环境以及生物学功能或结局，将EMT分为三种类型（图2）：（1）胚胎发育相关EMT（I型EMT）：胚胎的上皮细胞通过EMT过程实现由生长状态到运动状态的转变，进一步转移至其他部位，促进器官表型形成^[13]^。此型EMT不会引起纤维化以及侵袭性表型，所产生的间充质细胞往往可以进一步连续经历MET而产生次级上皮细胞；（2）组织再生及器官纤维化相关EMT（II型EMT）：在受损区域周围，上皮细胞经历EMT过程，迁移到伤口位置并进行增殖，从而生成纤维细胞，这些细胞对损伤修复和组织再生起到关键作用。该型EMT伴随着炎症反应，修复完成后炎症减轻，EMT进一步终止，慢性炎症的存在往往与组织的纤维化相关^[14]^；（3）肿瘤进展与转移相关EMT（III型EMT）：EMT的生物学特性赋予肿瘤细胞转移特性，增强侵袭性，侵入并定植在周围组织^[15]^。

**图2:**
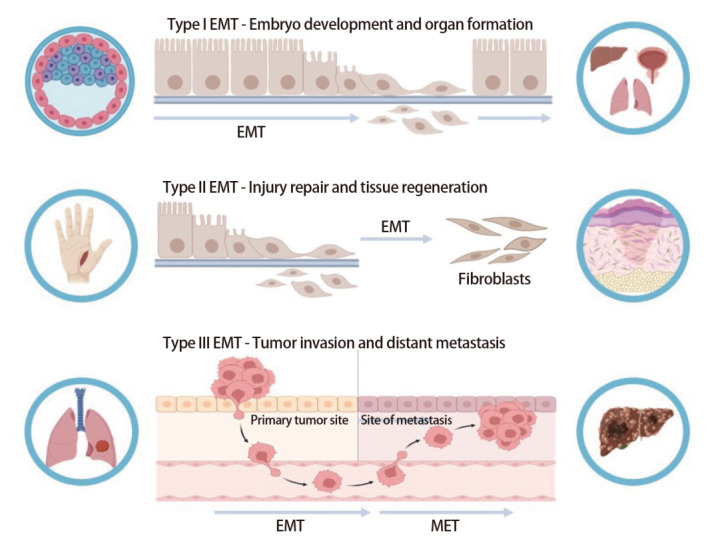
三种类型的EMT。I型EMT与胚胎发育和器官形成有关，细胞通过EMT过程实现运动与迁移，形成不同的器官。II型EMT与损伤修复和组织再生有关，损伤处的上皮细胞通过EMT转化为纤维细胞进行组织修复。III型EMT与肿瘤侵袭和远处转移有关，上皮型的肿瘤细胞通过EMT转化为可迁移的间充质细胞，迁移至新的组织器官后再通过MET过程恢复上皮细胞表型。

近期研究^[16]^表明，EMT的发生并非两个不同细胞群之间转化的二元化过程，在上皮细胞形态与完全间充质形态之间存在着不完全或混合EMT状态，即肿瘤细胞会在不同程度上经历EMT过程。EMT的不同过渡态的演变可能与基因调控网络、表观遗传以及肿瘤微环境（tumor microenvironment, TME）等多种因素相关^[17]^。

### 1.3 EMT发生的分子机制和调控

EMT诱导转录因子（epithelial mesenchymal transition-transcription factors, EMT-TFs）指能够直接抑制上皮细胞标志物基因的表达与直接诱导间充质细胞标志物基因表达的转录因子，多种EMT-TFs联合驱动EMT的发生。已被证实的EMT-TFs包括E盒结合锌指蛋白（zinc finger E-box binding homeobox, ZEB）家族（ZEB1/2）、蜗牛家族转录抑制因子1（Snail family transcriptional repressor 1, Snail1）、Snail2以及扭曲螺旋转录因子（twist family bHLH transcription factor, Twist）等^[18]^。Snail通过结合E-cadherin编码基因CDH1的启动子中的E盒来抑制其编码，ZEB1/2也能够通过该方式与E盒结合而抑制E-cadherin。ZEB1也可以通过募集染色质修饰子的方式促进Vimentin及N-cadherin的基因表达^[19]^。一项研究^[20]^证实存在不同表型的Slug（Snail2）可诱导正常乳腺干细胞与肿瘤起始细胞中不同的EMT程序。而Twist的表达与乳腺癌、肝癌等多种侵袭性肿瘤的表达有关，也是EMT的主要驱动因素之一^[18,21]^。

转化生长因子-β（transforming growth factor-β, TGF-β）细胞因子家族是EMT发生的最具特征的诱导因子^[22]^。TGF-β通路与EMT的发生密切相关，其介导作用涉及Smad分子依赖及非依赖性的两种通路实现。其中，Smad依赖性通路是经典机制，TGF-β与细胞表面受体复合物结合后，活化的受体复合物促使Smad2/3发生磷酸化，与Smad4结合形成复合物，该复合物进入细胞核与Snail1/2、ZEB等其他EMT-TFs相互作用，共同调控间充质基因的转录过程^[23,24]^。TGF-β可以通过磷酸化、乙酰化、泛素化等作用激活各种非经典通路^[25]^，也可以影响其他多种EMT相关信号通路的活动。除TGF-β通路外，无翅型MMTV整合位点家族（wingless type MMTV integration site family, Wnt）信号、Notch信号以及转化生长因子-α/核因子κB（transforming growth factor-α/nuclear factor kappa-B, TGF-α/NF-κB）等均为EMT发生的重要信号通路^[26⇓-28]^。EMT相关的信号通路并非独立发挥作用，其相互之间也可以影响，协同调节EMT的发生。

EMT的结果是产生具有间充质表型的可迁移细胞，但对于不同发生环境下的三类EMT，其诱导发生的分子机制存在一定的差异。I型EMT与胚胎着床及原肠胚的形成密切相关，主要由经典的Wnt信号途径、成纤维细胞生长因子（fibroblast growth factor, FGF）及骨形态发生蛋白（bone morphogenetic protein, BMP）等信号分子诱导，通过Snail等转录因子调控细胞黏附分子和细胞黏性蛋白的表达^[29]^。Wnt与FGFs共同调节原肠胚形成相关的EMT^[30,31]^，BMP诱导神经嵴细胞迁移的作用最显著^[6]^。II型EMT主要由TGF-β、血小板源性生长因子（platelet-derived growth factor, PDGF）等激活^[32]^，上调成纤维细胞特异蛋白-1（fibroblast specific protein-1, FSP-1）、α-平滑肌肌动蛋白（α-smooth muscle actin, α-SMA）等间质标志物。III型EMT发生在肿瘤细胞中，涉及TGF-β、Wnt、Notch及NF-κB等信号通路的异常激活和Snail、Slug、ZEB1、ZEB2及Twist等多种转录因子的改变^[26,27,33]^。

## 2 EMT与肺癌侵袭转移的关系

EMT是肿瘤进展恶化与侵袭转移的关键事件之一，其与肿瘤进展的关系已经在乳腺癌、前列腺癌及肝癌等中得到了证实^[33⇓⇓-36]^。肺癌是世界范围内癌症相关性死亡的首要原因，而导致肺癌患者死亡最主要的因素是原发肿瘤的侵袭转移与复发，存在微小病灶的早期肺癌患者术后复发与肿瘤细胞侵袭转移密切相关。近年来，多项研究^[37⇓-39]^也发现EMT在促进肺癌细胞侵袭和迁移中发挥重要作用。

一项回顾性队列研究^[40]^揭示了E-cadherin、Twist、Snail等EMT相关因子的表达是N0期非小细胞肺癌（non-small cell lung cancer, NSCLC）的重要预后因素，P-钙黏蛋白（P-cadherin）的高表达与NSCLC患者的肿瘤进展及不良预后也显著相关^[37]^，这与Twist和Snail在癌症中预后作用研究的系统评价研究结果一致^[38]^。Twist还可能通过改变EMT标志物的表达促进NSCLC的缺氧浸润及转移^[39]^。更早的研究^[41]^也指出Twist、Slug和Foxc2表达对I期NSCLC根治性切除术后具有预后作用。

EMT通过改变细胞骨架结构促进肺癌细胞的侵袭与转移。E-cadherin的下调与N-cadherin的上调影响了肿瘤干细胞（cancer stem cells, CSCs）间的细胞连接，促进肿瘤细胞的侵袭与迁移。内吞接头蛋白Epsin-3的低表达可通过抑制EMT相关通路而增加E-cadherin的表达，进一步抑制肺腺癌细胞的转移与侵袭能力^[42]^。此前的研究^[43]^也证实在小鼠肺腺癌模型中E-cadherin的缺失加速了肿瘤的进展与转移。细胞骨架结构蛋白Claudin-3的表达已被证明与肺鳞状细胞癌的预后显著关联，有研究^[44]^表明肺鳞癌中Claudin-3的表达与E-cadherin及Vimentin显著关联，由此说明Claudin-3归因于EMT过程，通过E-cadherin及Vimentin影响肺鳞癌的进展与转移。有研究^[45]^也发现，低E-cadherin和高N-cadherin表达在鳞状细胞癌中显著高于腺癌，肺鳞癌细胞与腺癌细胞EMT过程中细胞骨架的改变及EMT的结局存在差异，这可能与两种细胞不同的侵袭转移特点有关。

基质金属蛋白酶（matrix metalloproteinases, MMPs）家族可诱导EMT的发生。一方面，这类酶可以切割E-cadherin，降解细胞外基质，为肿瘤细胞的迁移侵袭提供通道；另一方面，MMPs家族可以通过表皮生长因子受体（epidermal growth factor receptor, EGFR）信号通路诱导EMT，并通过降解的细胞外基质（extracellular matrix, ECM）释放生长因子，促进新生血管形成^[46]^。研究^[47]^表明，抗肿瘤药物沙利霉素（Salinomycin）能通过减少MMP-2和MMP-9的表达来抑制TGF-β1触发的EMT，进而阻碍肺癌细胞的迁移与侵袭过程。在小鼠肺上皮细胞中，MMPs诱导的Ras相关C3肉毒杆菌毒素底物1b（Ras-related C3 botulinum toxin substrate 1b, Rac1b）表达对EMT有激活作用。近期有研究^[48,49]^指出MMPs可通过多种通路介导香烟烟雾诱导的气道上皮EMT。在肺癌细胞EMT的研究^[50]^中，靶向MMPs基因的小向导RNAs（small guide RNAs, sgRNAs）可明显抑制肿瘤细胞的迁移活性。

## 3 EMT与肺癌耐药的关系

靶向治疗、化疗与免疫治疗在肺癌的治疗中处于重要地位。然而，多数的肺癌仍面临耐药性问题，导致疾病的进展甚至死亡。EMT过程与肺癌细胞的耐药性密切相关，但其潜在机制尚不完全清楚，TME及各种细胞因子的刺激以及细胞内各种复杂的EMT相关信号转导通路的激活或下调，最终导致了肺癌细胞的耐药（图3）。

**图3:**
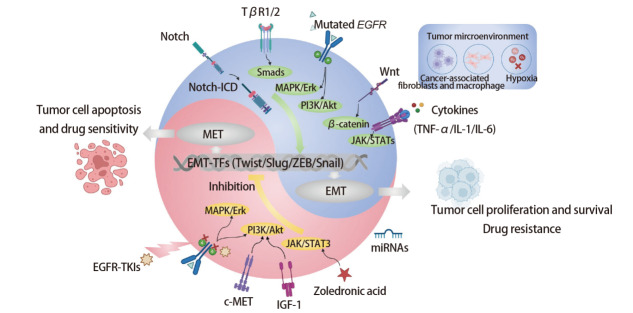
EMT与肺癌耐药的机制。蓝色部分表示EMT导致肺癌耐药的相关机制。Notch释放其胞内段结构域，TGF-β介导Smads信号通路，EGFR介导MAPK/Erk通路与PI3K/Akt通路，Wnt介导β-catenin通路，最终介导激活EMT-TFs，促进肿瘤细胞的增殖存活，增加其耐药性。肿瘤微环境中的成纤维细胞和巨噬细胞释放的TNF-α、IL-1、IL-6以及缺氧产生的细胞因子等通过JAK/STATs通路也可介导EMT-TFs的激活。红色部分表示EMT导致肺癌耐药相关的治疗靶点。EGFR-TKIs通过抑制EGFR的磷酸化过程进而抑制EGFR介导的下游信号转导，抑制肿瘤细胞的EMT过程，促进MET发生，导致肿瘤细胞凋亡及药物敏感。c-Met及IGF-1通路介导PI3K/Akt等也可降低EMT诱导的EGFR-TKIs耐药。唑来膦酸及miRNAs等也可介导下游信号通路促进耐药肺癌细胞重新敏感。

### 3.1 EMT介导的肺癌耐药机制

第一，EMT通过药物靶点改变与信号通路重构促进肺癌细胞对药物诱导的细胞凋亡的抵抗。EMT导致药物靶点的改变，使得针对上皮表型肺癌细胞的药物难以有效结合并发挥作用。例如，EGFR、间变性淋巴瘤激酶（anaplastic lymphoma kinase, ALK）、c-ros原癌基因1-受体酪氨酸激酶（c-ros oncogene 1, receptor tyrosine kinase, ROS1）等上皮型癌细胞特有的受体在EMT过程中发生下调或功能丧失，而间充质型癌细胞则可能表达其他类型的受体或信号分子，从而使得药物在EMT后的癌细胞中疗效大减^[51]^。EMT还伴随着细胞内信号通路的重构，磷脂酰肌醇3激酶/蛋白激酶B（phosphatidylinositol 3-kinase/protein kinase B, PI3K/Akt）、丝裂原活化蛋白激酶/细胞外信号调节激酶（mitogen-activated protein kinase/extracellular signal-regulated kinase, MAPK/Erk）、Notch和Wnt等通路被激活或抑制^[52,53]^。研究^[54]^表明，PI3K/Akt通路的持续激活可以抑制凋亡相关蛋白如Caspase的活性，从而阻止细胞凋亡的发生。同时，MAPK/Erk通路的激活也可以促进癌细胞的增殖与耐药性的产生^[53]^。

第二，EMT与CSCs分化特性密切相关。CSCs是维持肿瘤细胞生长与自我增殖的干细胞，转移性肿瘤干细胞（metastatic cancer stem cells, MCSCs）是其中的一个亚群，可接受远端器官环境的基质信号并从原发肿瘤的边界逃逸。EMT过程与CSCs特性的获得密切相关，可增加MCSCs的侵袭性表型转化，促使其从原发部位侵入周围组织、进入循环并在远端组织中定植形成转移灶^[55]^。同时，CSCs表达与间充质细胞相同的EMT-TFs，CSCs和EMT的相关通路之间具有广泛的相互作用，共同促进肿瘤细胞的耐药^[56]^。因此，EMT介导的肺癌耐药可以依赖与CSCs相同的机制。CSCs表面过表达的各类ABC家族转运蛋白（如ABCB1、ABCG2）等已经被证明可以导致化疗耐药，另外，CSCs通过降低化疗引起的细胞内活性氧（reactive oxygen species, ROS）的产生以及增加DNA自我修复倾向来产生化疗耐药。在TGF-β诱导的EMT中也观察到了ABCB1、ABCC1等ABC家族转运蛋白的上调^[57]^，由此说明EMT可能与CSCs有同样的耐药机制。

第三，TME的改变也是EMT引起肿瘤耐药的因素之一。在低氧条件下，缺氧诱导因子-α（hypoxia-inducible factor-α, HIF-α）对NF-κB通路的激活以及对PI3K、Wnt等通路的激活均与CSCs的自我更新以及EMT表型转变密切相关。另外，TME的基质细胞如癌症相关成纤维细胞（cancer-associated fibroblasts, CAFs）、巨噬细胞等通过白介素-6（interleukin-6, IL-6）等细胞因子促进EMT的发生，从而促进肿瘤的耐药^[58]^。

### 3.2 EMT介导的肺癌靶向治疗耐药

肺癌的靶向治疗是针对肺癌患者已明确的致癌点位进行针对性用药，使肿瘤细胞特异性死亡的一种精准治疗方法，主要依赖于癌细胞和正常细胞在分子层面上的差异，通过识别并作用于特定的分子“靶点”来阻止癌细胞的生长或帮助免疫系统更好地识别癌细胞^[59]^。EGFR基因是NSCLC的代表性驱动基因，占所有NSCLC病例的10%-15%^[60]^。EGFR-酪氨酸激酶抑制剂（EGFR-tyrosine kinase inhibitors, EGFR-TKIs）是肺癌靶向治疗的代表性药物，适用于EGFR基因敏感突变的局部晚期或转移性NSCLC患者^[61]^。

EGFR-TKIs主要通过阻断EGFR内的酪氨酸激酶活性，有效阻止下游信号通路（如PI3K通路）的磷酸化过程，从而抑制肿瘤细胞活性并促进凋亡^[62]^。近年来，研究^[81]^表明间充质细胞表型可以诱导癌细胞产生EGFR-TKIs耐药。关于耐药持久性（drug tolerant persister, DTP）细胞产生的研究^[5]^发现，第一代与第三代的EGFR-TKIs 耐药可能与肺癌细胞间充质表型的获得有关。一项关于76基因EMT特征验证的研究^[63]^也表明上皮性的NSCLC细胞表达激活EGFR通路的生物标志物，同时间充质细胞对抑制EGFR的化合物更具抗性。E-cadherin等上皮细胞标志物的缺失提示EMT过程的发生，当E-cadherin表达缺失时肿瘤细胞对EGFR-TKIs的耐药性增加，而恢复E-cadherin的表达则可重新增加其对TKIs的敏感性^[64]^。

有关EFGR-TKI获得性耐药的研究^[82]^指出，Twist、ZEB1、Slug及Notch等EMT相关转录因子均在介导NSCLC细胞对TKIs耐药过程中起到重要的调控作用。ZEB1可通过E-cadherin和Vimentin等表达的改变介导NSCLC对EGFR-TKIs治疗的获得性耐药。一项整合组学研究^[65]^也指出Cadherin-3可作为肺癌EGFR-TKIs治疗的生存预测因子和早期监测标志物。

在EGFR-TKIs耐药的肺癌细胞中，Notch-1激活可以促进EMT并增加Snail和Vimentin的表达，由此说明吉非替尼耐药是Notch激活EMT的继发结果^[28]^。此外，其他酪氨酸激酶受体相关信号转导通路也可能通过旁路机制引起EMT相关的EGFR-TKIs耐药。例如，肝细胞生长因子/间质表皮转化因子（hepatocyte growth factor/cellular-mesenchymal epithelial transition factor, HGF/c-Met）和胰岛素样生长因子1（insulin-like growth factor 1, IGF-1）通路的双重联合抑制效应可显著抑制膜联蛋白A2（Annexin A2）的表达，并显著降低癌症相关成纤维细胞诱导的EMT与EGFR-TKIs耐药^[66]^。

在发生EMT的肺癌细胞中，EGFR的表达及活性会发生变化，而其下游信号分子如Akt激活，这种激活可能通过非EGFR依赖性机制发生，进而介导PI3K/Akt信号通路的持续激活。该通路的持续上调可以抑制肿瘤细胞的凋亡，促进细胞增殖和迁移，从而增强肺癌细胞的耐药性和侵袭性。研究^[52]^表明IL-1β激活的MEK/Erk信号通路可与PI3K/Akt信号通路协同促进肿瘤细胞EMT的发生。此外，近期也有研究^[67]^报道了Janus激酶/信号转导和转录激活因子3（Janus kinase/signal transducer and activator of transcription 3, JAK/STAT3）信号通路与肿瘤细胞EMT密切相关，唑来膦酸（Zoledronic acid）可通过该信号通路介导逆转EMT过程，促进吉非替尼耐药的肺癌细胞重新敏感，成为潜在的耐药性治疗靶点之一。

### 3.3 EMT介导的肺癌化疗耐药

关于醛酮还原酶1C3（aldo-keto reductase family 1 member C3, AKR1C3）与肿瘤耐药的研究^[68]^发现，AKR1C3可通过诱导小细胞肺癌（small cell lung cancer, SCLC）中的EMT与新生血管生成而导致耐药。靶向人类肺腺癌转移相关转录本（metastasis associated lung adenocarcinoma transcript, MALAT）的miR-145可通过降低EMT过程而降低NSCLC对顺铂的耐药性^[69]^。经顺铂长期处理而耐药的肺癌细胞及其亲本细胞中的上皮标志蛋白与间充质标志蛋白测定表明，耐药细胞的E-cadherin表达降低，N-cadherin、Vimentin与Snail的表达升高，这表明持续性顺铂刺激可通过EMT途径导致肿瘤细胞耐药^[70]^，与更早的一项顺铂耐药的肺癌细胞EMT表型分析的研究^[71]^结果类似。此外，多项研究^[72,73]^也表明培美曲塞、吉西他滨及紫杉醇类等化疗药物的耐药与EMT有关。

### 3.4 EMT介导的肺癌免疫治疗耐药

免疫治疗通过激活患者自身的免疫系统来识别和攻击癌细胞，已成为肺癌治疗的重要手段之一^[74]^。然而，部分患者在接受免疫治疗后仍会出现疾病进展或复发，这可能与EMT介导的免疫治疗耐药有关^[75]^。

在EMT过程中，癌细胞表面的分子标志物发生变化，导致免疫细胞难以有效识别和攻击。在EMT过程中，癌细胞表面的主要组织相容性复合体（major histocompatibility complex, MHC）分子和共刺激分子的表达可能下调，导致免疫细胞难以识别并攻击这些细胞，促进了肺癌细胞的免疫逃逸^[75]^。间充质型肺癌细胞还可能表达免疫抑制性分子如细胞程序性死亡配体1（programmed cell death ligand 1, PD-L1），进一步抑制免疫细胞的活性和功能^[76]^。此外，EMT过程伴随着TME的改变，癌细胞与周围基质细胞、免疫细胞等发生相互作用，形成有利于癌细胞生长和逃逸的微环境^[75]^。例如，CAFs等基质细胞可以通过分泌细胞因子和趋化因子等促进癌细胞的增殖、迁移和侵袭，同时抑制免疫细胞的活性和功能^[77]^。这些变化不仅会加剧癌细胞的恶性表型，还可能降低免疫治疗的效果。

### 3.5 EMT介导的肺癌耐药的研究展望

针对EMT相关的肺癌耐药，当前研究正逐步揭示其复杂机制，并探索潜在的解决策略。对EMT过程的抑制不仅有望恢复肿瘤细胞对化疗和靶向治疗的敏感性，还可能为肺癌治疗开辟新的途径。

EMT相关的分子标志物如E-cadherin、Vimentin、ZEB1等的表达水平变化，可能预示着肺癌细胞对特定治疗的敏感性或耐药性。同时，microRNA、EMT-TFs相关因子与肺癌耐药的研究也广泛开展，以上生物标志物有助于揭示EMT介导耐药的分子机制，还可能成为预测耐药性和指导个性化治疗的有力工具。

针对EMT有关的肺癌耐药，目前已有一些潜在的解决方案。对EMT过程的抑制可以恢复肿瘤细胞对化疗和靶向治疗的敏感性。关于EMT介导的肿瘤耐药相关研究，重点是通过EMT抑制剂来减少肺癌细胞的耐药性，改善治疗效果以及临床预后。一项乳腺癌的研究^[78]^通过人工智能方法筛选通过microRNA介导的EMT抑制剂以解决肿瘤的耐药性问题。EGFR/PIM1的双阻断也可协同逆转奥希替尼耐药的NSCLC^[79]^。EMT标志物的靶向治疗药物也在研发中，针对TGF-β的治疗策略目前处于实验测试或早期临床试验阶段^[80]^。除此之外，将化疗药物与靶向药物联合使用，或者将免疫治疗与靶向药物联合使用，可以通过双重机制发挥作用，有效抑制肿瘤细胞增殖并促进其凋亡，从而延缓病情进展。

## 4 结语

综上所述，EMT与肺癌耐药性之间存在着密切的关系。EMT不仅参与肺癌的发生、发展和转移过程，还在肺癌耐药性的形成中发挥重要作用。然而，目前EMT如何通过调节靶点表达、DNA修复及表观遗传等过程影响耐药性的确切机制仍需进一步深入研究。缺乏大样本、多中心的临床研究来验证EMT相关分子标志物与肺癌耐药性的直接关联。因此，深入研究EMT的分子机制，开发有效的逆转策略，有效解决肺癌耐药问题，可以为肺癌患者提供更加精准和有效的治疗选择，为患者带来更好的临床预后。
